# *oriC*-encoded instructions for the initiation of bacterial chromosome replication

**DOI:** 10.3389/fmicb.2014.00735

**Published:** 2015-01-06

**Authors:** Marcin Wolański, Rafał Donczew, Anna Zawilak-Pawlik, Jolanta Zakrzewska-Czerwińska

**Affiliations:** ^1^Department of Molecular Microbiology, Faculty of Biotechnology, University of WrocławWrocław, Poland; ^2^Department of Microbiology, Ludwik Hirszfeld Institute of Immunology and Experimental Therapy, Polish Academy of SciencesWrocław, Poland

**Keywords:** *oriC*, DnaA, initiation of chromosome replication, orisome, replication regulation, regulatory proteins, bacteria

## Abstract

Replication of the bacterial chromosome initiates at a single origin of replication that is called *oriC*. This occurs via the concerted action of numerous proteins, including DnaA, which acts as an initiator. The origin sequences vary across species, but all bacterial *oriCs* contain the information necessary to guide assembly of the DnaA protein complex at *oriC*, triggering the unwinding of DNA and the beginning of replication. The requisite information is encoded in the unique arrangement of specific sequences called DnaA boxes, which form a framework for DnaA binding and assembly. Other crucial sequences of bacterial origin include DNA unwinding element (DUE, which designates the site at which *oriC* melts under the influence of DnaA) and binding sites for additional proteins that positively or negatively regulate the initiation process. In this review, we summarize our current knowledge and understanding of the information encoded in bacterial origins of chromosomal replication, particularly in the context of replication initiation and its regulation. We show that *oriC* encoded instructions allow not only for initiation but also for precise regulation of replication initiation and coordination of chromosomal replication with the cell cycle (also in response to environmental signals). We focus on *Escherichia coli*, and then expand our discussion to include several other microorganisms in which additional regulatory proteins have been recently shown to be involved in coordinating replication initiation to other cellular processes (e.g., *Bacillus, Caulobacter, Helicobacter, Mycobacterium*, and *Streptomyces*). We discuss diversity of bacterial *oriC* regions with the main focus on roles of individual DNA recognition sequences at *oriC* in binding the initiator and regulatory proteins as well as the overall impact of these proteins on the formation of initiation complex.

## Introduction

**Figure 1:**
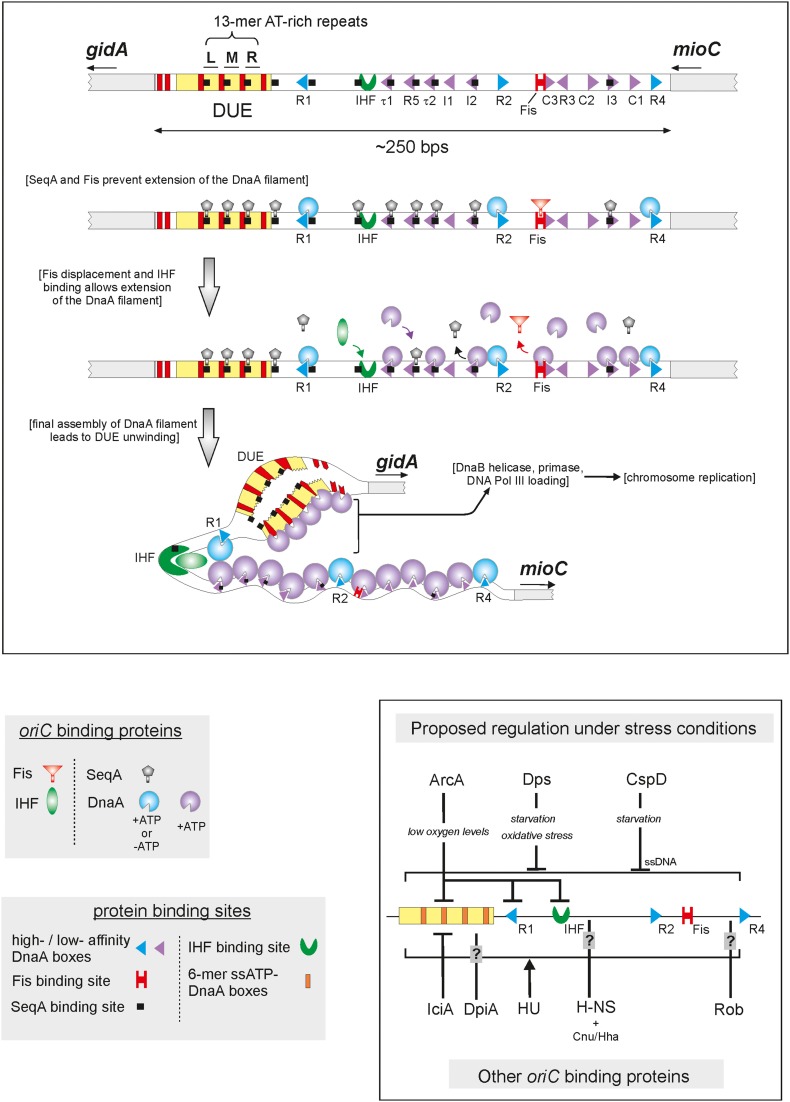
**A model of initiation replication and its regulation in *E. coli* by origin binding proteins (oriBPs)**. Large panel presents assumed sequence of events during the replication initiation and roles of particular oriBPs. The unwound DUE is accessible to the replication proteins complex (e.g., helicase DnaB, primase, and DNA Pol III). Small panel shows additional oriBPs divided in two subgroups, those involved in alternative scenarios that may occur under environmental stress conditions (upper part of the panel) and others, including those of unknown function (bottom part of the panel). Triangles' directions represent orientations of DnaA binding sites. Nucleotide bound status of DnaA is represented by blue and violet incomplete circles. Small arrows below gene names indicate gene orientations. In the small panel, different types of vertical lines represent type of action, activation (arrow), inhibition (bar-headed line) or unknown (question mark line). Horizontal lines indicate unspecific binding to *oriC*.

In contrast to the situation in Eukaryotes, chromosomal replication in bacteria begins at a single site on the chromosome: the origin of replication (*oriC*) (Leonard and Méchali, 2013). Using various *in silico* approaches (Mackiewicz et al., 2004; Gao et al., 2013), researchers have predicted the locations of the *oriCs* for more than 1500 bacterial chromosomes. However, *in vivo* replication activity has been confirmed for only a dozen such origins. Over the last 30 years, researchers have made considerable progress in understanding the mechanisms of replication initiation, particularly the organization and function of the *oriC* region in *Escherichia coli* (Figure 1), which is a model microorganism for the study of chromosomal replication (for reviews, see references, Fuller et al., 1984; Hwang and Kornberg, 1992; Messer, 2002; Kaguni, 2006, 2011; Leonard and Méchali, 2013). These studies have shown that replication is initiated through the cooperative binding of the initiator protein, DnaA, to multiple DnaA-recognition sites (boxes) within the *oriC* region. This triggers separation of the DNA strands at the AT-rich DNA unwinding element (DUE), providing an entry site for helicase and later on the other enzymes (e.g., primase and DNA Pol III) that are responsible for DNA synthesis.

Comparative sequence analysis has demonstrated that the origin regions with confirmed *in vivo* functions differ in their sequences, organizations, and sizes, with only closely related organisms exhibiting fairly high overall similarities in their *oriC* sequences (Jakimowicz et al., 1998; Zawilak-Pawlik et al., 2005). In addition to a diverse repertoire of DnaA boxes, *oriC* regions also include various binding sites for accessory and regulatory proteins.

Chromosomal replication is mainly controlled at the initiation step (Mott and Berger, 2007; Zakrzewska-Czerwińska et al., 2007; Katayama et al., 2010; Leonard and Grimwade, 2010, 2011; Skarstad and Katayama, 2013). Therefore, the activities of the *oriC* region must be tightly regulated to guarantee that chromosomal DNA is entirely replicated only once per cell cycle. This is achieved by regulating the accessibility of *oriC* to DnaA, which occurs mainly via the binding of other proteins. Additionally, replication initiation is regulated by the modulation of DnaA protein activity.

The main goal of this review is to highlight the diversity of *oriC* regions in the context of replication regulation and the bacterial cell cycle. We focus on how *oriC* regions have adjusted to coordinate the regulation of chromosome replication and the progression of the cell cycle. We postulate that *oriC* regions encode species- and genus-specific instructions for the orderly binding of DnaA and other proteins responsible for forming the functional initiation complex (orisome) and/or regulating the assembly of this complex.

## *oriC* chromosomal localisation and nucleotide sequence are not strictly conserved

**Figure 2:**
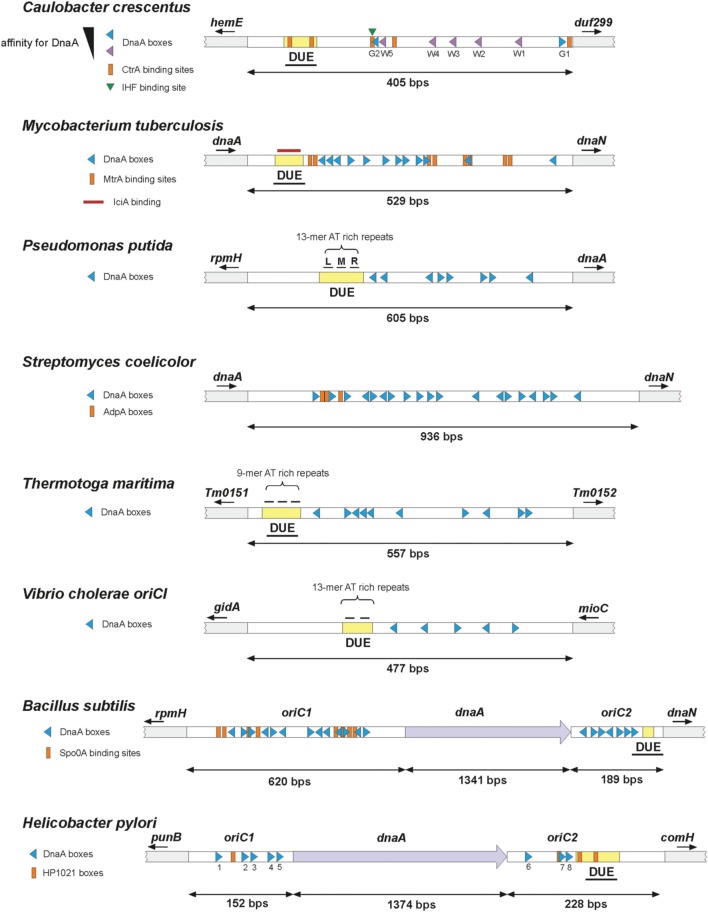
**The structures of selected bacterial origins**. Continuous origin – upper part of the panel; bipartite origin – lower part of the panel. OriBP regulators' binding sites are presented if described in the literature. DUE – DNA unwinding element, underlined DUE indicates experimentally confirmed unwinding. Triangles' directions represent orientations of particular DnaA boxes. Small arrows below gene names indicate gene orientations.

The *oriC* regions are usually flanked by the *dnaA* gene and sometimes also the *dnaN* gene (Figure 2). These genes encode two pivotal proteins for initiating and continuing replication in the bacterial chromosome: DnaA, which is described above, and DnaN, which encodes a beta sliding clamp responsible for the processivity of DNA polymerase III. In linear chromosomes, such as those of *Streptomyces coelicolor* and (presumably) *Borrelia burgdorferi, oriC* is located in the center of the chromosome (Zakrzewska-Czerwińska and Schrempf, 1992; Mackiewicz et al., 2004). The region of gene synteny around *oriC*, which includes the highly preserved gene cluster of *rnpA-rpmH-dnaA-dnaN-recF-gyrB-gyrA*, is conserved in some (even distantly related) bacterial species. For a long time, the presence of these genes was assumed to mark the chromosomal localization of *oriC* (Ogasawara et al., 1991; Briggs et al., 2012). However, in many bacteria, including the model bacterium, *E. coli*, the *oriC* region is located in another gene context, indicating that this conserved gene cluster is not important for *oriC* function (Briggs et al., 2012). The localization of *oriC* is not random either as recent studies have indicated that the *oriC*-proximal gene context is conserved in certain group(s) of bacteria (e.g., genus or species). This is thought to enable a robust response to unfavorable conditions by allowing bacteria to increase the gene dosage in response to stress-induced initiation events (Moriya et al., 2009; Slager et al., 2014).

Interestingly, a few obligate endosymbiotic bacteria, such as *Wigglesworthia glossinidia, Blochmannia floridanus*, and *Candidatus Endolissoclinum faulkneri*, lack the *dnaA* gene (Akman et al., 2002; Gil et al., 2003; Mackiewicz et al., 2004; Kwan and Schmidt, 2013). It is not known how these bacteria initiate chromosome replication and whether their *oriC*s resemble the bacterial origins characterized to date. Indeed, it has been postulated that the typical DnaA box cluster might not exist/be functional in these bacteria.

The nucleotide sequences of the *oriC* regions are highly diverse across unrelated species. Thus, they are not active in or interchangeable between unrelated bacteria (O'Neill and Bender, 1988), and sequence homology alignment of *oriC* is not used to identify unknown origins in bacterial genomes. In closely related bacteria, however, the sequence homology (and thus the organization) of the entire *oriC* region might be high enough to enable the *oriC* region from one species to autonomously replicate in another species (Harding et al., 1982; Takeda et al., 1982; Zyskind et al., 1983; Roggenkamp, 2007). It might also be possible to substitute the origin of one species with that of a related species, as was shown in the successful substitution of the *E. coli oriC* for the *Vibrio cholerae* origin of replication in chromosome I (Demarre and Chattoraj, 2010; Koch et al., 2010). It is important to note that when bacteria possess two or more chromosomes, only one undergoes replication initiated by the DnaA protein at the origin typical for bacterial chromosomes (e.g., the *V. cholerae oriC*I). The replication origin on the other chromosome is plasmid like, and it is activated by initiators that lack homology to DnaA (e.g., *V. cholerae oriC*II is initiated by a RctB protein) (Egan and Waldor, 2003; Duigou et al., 2006). Such diversification avoids the need for the chromosomes to compete for initiator proteins, and yields better control of their separate but coordinated replications (Duigou et al., 2006; Jha et al., 2012; Baek and Chattoraj, 2014).

## The *oriC* region can be continuous or bipartite and consists of functional modules

A replication origin may be continuous or bipartite. Most of the bacteria studied to date contain a continuous *oriC* region that includes all of the functional modules within a single intergenic region (Figure 2). The divided origins, in contrast, are composed of two subregions, each of which contains a cluster of DnaA boxes, and one of which harbors the DUE region (Figure 2). They also differ in length: the continuous origins range from ~250 (*E. coli*) to ~950 bps (*Streptomyces*) (Zakrzewska-Czerwińska and Schrempf, 1992; Jakimowicz et al., 1998), while the bipartite origins are longer, up to ~2000 bps, because they contain a spacer gene (usually *dnaA*) between the *oriC* subregions (Figure 2; see below for mollicute origins). We do not yet know why some origins are split. Experimental data have shown that the spacer is important *per se*, although it may be altered to some extent without the loss of *oriC* activity. For example, a study showed that the spacer linking the *oriC*1 and *oriC*2 regions in *Bacillus subtilis* can be shortened (Moriya et al., 1992). Up until recently, the bipartite origin was assumed to be characteristic of a few Gram-positive bacteria (*B. subtilis* and *Streptococcus pyogenes*) and *Mollicutes* (*Mycoplasma* sp., *Spiroplasma* sp.) (Krause et al., 1997; Moriya et al., 1999; Suvorov and Ferretti, 2000; Lee et al., 2008; Briggs et al., 2012). However, bipartite origins were also recently identified in Gram-negative bacteria (e.g., *Helicobacter pylori*) (Donczew et al., 2012, 2014b), suggesting that bipartite origins might be more common than previously thought in diverse bacterial species. The origins of mollicutes were reported to have unusual properties, with interchangeability observed between species having divergent organizations of their *oriC*s (e.g., differences in the number, orientation and sequence of the DnaA boxes and/or the localization of the AT-rich regions) (Lartigue et al., 2003; Lee et al., 2008). The origins in mollicutes should be analyzed with caution, however, because they were identified solely by the minichromosome approach and no detailed characterization has yet been performed.

The study of the bacterial replication origin has progressed beyond the characterization of bacterial DnaA proteins, their assembly onto *oriC*, and the modes of initiation complex (orisome) formation in various bacterial species. However, it remains difficult to interpret the differences in bacterial *oriC*s in the context of their activities. As discussed above, the *oriC* regions characterized to date are quite diverse in terms of their chromosomal loci, genetic contexts, nucleotide sequences, lengths and continuities. However, they are all composed of three basic functional modules: a cluster (or multiple clusters) of DnaA boxes, the DUE region, and other sequences that are recognized by regulatory proteins. These modules constitute the central management system for orisome formation, but their numbers and relative localizations vary across organisms (Figure 2).

In each species, this organized information provides a perfect molecular scaffold for DnaA oligomerization, controls DNA opening, and regulates the initiation of chromosomal replication. These processes are detailed in the following sections.

## The arrangements of the DnaA boxes and “DUE” are crucial for *oriC* activity

The role of particular *oriC* modules has been widely studied in *E. coli*, providing a comprehensive example of a replication initiation mechanism and its interplay with cellular regulatory circuits. The DnaA boxes constitute a framework for the binding of DnaA monomers, which interact with *oriC* to form a structure that is able to disturb the DNA double-helix. The unique layout of low- and high-affinity DnaA boxes in *E. coli oriC* regulates the formation of a specific DnaA oligomer, which (according to the current model) adopts the structure of a right-handed helical filament to directly stimulate DNA unwinding (Erzberger et al., 2006; Zorman et al., 2012). The particular DnaA molecules involved in the filament introduce a bend in the DNA helix, which is gradually wrapped around the filament's outer surface (Fujikawa et al., 2003; Erzberger et al., 2006). The final complex introduces a superhelical tension in the DNA helix; this is likely to be focused in the DUE region, and triggers the initial unwinding (Erzberger et al., 2006). In the subsequent steps, the ATP-DnaA oligomer is believed to bind the newly formed single-stranded DNA segments, stabilizing and stretching them to promote further extension of the initiation bubble (Duderstadt et al., 2011; Ozaki and Katayama, 2012). The formation of a similar helical DnaA oligomer was recently shown for *B. subtilis* in the presence of both single-stranded and double-stranded DNA (Scholefield et al., 2012). Scholefield et al. suggested that separate oligomers may be involved in the unwinding and subsequent stabilization of the single-stranded DUE in this case, but we do not yet understand the mechanism underlying oligomer formation in *B. subtilis* in terms of the bipartite structure of its origin or the subsequent steps of the initiation process. It also remains to be seen whether other bacterial initiation complexes involve the formation of similar higher-order structures. Nevertheless, it is plausible that the formation of a DnaA-containing oligomer is essential for the unwinding of DNA at the DUE region, and is thus a common feature of all bacterial origins.

The number of DnaA boxes in the studied origins ranges from five in *Pseudomonas aeruginosa* or *V. cholerae* to 19 in *S. coelicolor* (Figure 2). In most cases, these DnaA boxes are asymmetrical nine-nucleotide-long specific motifs (with the exception of the 12-nucleotide boxes found in *Thermotoga maritima*), whose exact sequences, numbers and layouts reflect the diversity of the various organisms. DnaA boxes from different bacteria do, however, share a common core sequence (Table 1). No analyzed origin contains boxes, which deviate by more than two mismatches from the so-called “perfect” box sequence (i.e., that which binds with the highest affinity) of *E. coli* (TTATCCACA), with the exception of that of *T. maritima*. In closely related organisms, the high-affinity box sequence is conserved, as seen in *E. coli, V. cholerae, Pseudomonas putida* and *P. aeruginosa*, which all belong to a branch of the γ-proteobacteria (Yee and Smith, 1990; Weigel et al., 1997; Egan and Waldor, 2003) (Table 1). Interestingly, even *B. subtilis*, which is evolutionarily distant from *E. coli*, shares the same conserved “perfect” box sequence (Fukuoka et al., 1990). As noted above, the “perfect” DnaA box in other species almost always differs from this *E. coli* DnaA box by only one or two nucleotides. In Actinomycetes (*M. tuberculosis* and *S. coelicolor*), which are considered high-GC organisms, the “perfect” DnaA box contains G or C at the third position: TT(G/C)TCCACA (Jakimowicz et al., 2000; Zawilak et al., 2004; Tsodikov and Biswas, 2011). Similarly, *Caulobacter crescentus* G-boxes (see Figure 2 and Table 1) differ from the *E. coli* consensus sequence by a single nucleotide (in this case in the second position; TGATCCACA) (Shaheen et al., 2009). Among the studied origins, the nine-nucleotide DnaA boxes most distant from that of *E. coli* are found in *H. pylori*, which contain two mismatches (at the second and fifth positions; TCATTCACA) with respect to the “perfect” *E. coli* sequence (Zawilak et al., 2001; Donczew et al., 2014b). An interesting exception from this general rule is the *T. maritima* origin, where ten 12-nucleotide DnaA boxes were identified (consensus DnaA box: AAACCTACCACC) (Ozaki et al., 2006). As *T. maritima* is one of the most ancient bacteria, it has been proposed that its DnaA boxes may resemble a sequence recognized by the initiator protein in a last common ancestor of the unicellular organisms (Ozaki et al., 2006). Indeed, the *T. maritima* DnaA box sequence shares some similarity to the ORC-binding sites in *Saccharomyces cerevisiae* (TAAACATAAAA) and the Orc1/Cdc6-binding sequences in Archaea (e.g., in *Methanothermobacter thermoautotrophicus* – TTACAGTTGAAA) (Ozaki et al., 2006). Thus, it is probable that the *E. coli*-like nine-mer DnaA box sequence has evolved from this original 12-nucleotide sequence, becoming shortened to nine nucleotides at some point. The last six nucleotides of the *T. maritima* consensus sequence (ACCACC) are similar to the corresponding part of the nine-nucleotide DnaA boxes. The importance of this six-nucleotide motif as an integral part of a bacterial DnaA box is further supported by the recent study of the *C. crescentus oriC*, where five six-nucleotide boxes (termed W-boxes) were identified in addition to the two known nine-nucleotide high-affinity G-boxes (Taylor et al., 2011). The W-box consensus sequence is TCCCCA, which deviates from the last six nucleotides of the *E. coli*-like box at the fourth position, and shows a very weak but detectable binding by the DnaA protein. Researchers have also identified atypical six-nucleotide-long DnaA boxes in *E. coli*; located directly in the DUE region and bound only in a single-stranded form, this consensus sequence (AGATCT) represents an alternative type of DnaA-recognized sequence (albeit so far exclusive for *E. coli*) (Speck and Messer, 2001).

**Table 1:** **Sequences of high-affinity DnaA boxes from various bacteria**.

**Organism**	**High-affinity DnaA box sequence**
*Escherichia coli*	TTATCCACA
*Vibrio cholerae*	TTATCCACA
*Pseudomonas putida*	TTATCCACA
*Bacillus subtilis*	TTATCCACA
*Caulobacter crescentus*	TGATCCACA
*Helicobacter pylori*	TCATTCACA
*Mycobacterium tuberculosis*	TTGTCCACA
*Streptomyces coelicolor*	TTGTCCACA
*Thermotoga maritima*	AAACCTACCACC

The origins of closely related bacteria may be interchangeable to some degree between species, as shown *in vivo* (Koch et al., 2010) and *in vitro* (Jiang et al., 2006) for members of the γ-proteobacteria branch. Among evolutionarily distant bacteria, the situation is more complicated. Considering the similarity of DnaA boxes from different organisms, it is not surprising that the DnaA protein is often able to recognize DnaA boxes and/or whole *oriC* regions in heterologous systems *in vitro*, albeit often with a lower affinity and/or specificity. For example, *S. coelicolor oriC* (*ScoriC*) is bound efficiently by the *M. tuberculosis* and *E. coli* DnaA proteins, but neither protein was able to bend the *ScoriC* structure in the manner of the native DnaA protein (Jakimowicz et al., 2000; Zawilak-Pawlik et al., 2005). Furthermore, the origins of *S. coelicolor, M. tuberculosis* and *H. pylori* were not found to be active in *E. coli* cells, even though they are efficiently bound by the *E. coli* DnaA *in vitro*. Interestingly, the *H. pylori* DnaA interacts very poorly with the *E. coli oriC*, indicating that even *in vitro* DnaA/oriC systems from different species may be interchangeable in one setting (i.e., *E. coli* DnaA/*H. pylori oriC*) but not the other (i.e., *H. pylori* DnaA/*E. coli oriC*) (Zawilak-Pawlik et al., 2005). Furthermore, the DnaA proteins of *E. coli* and *B. subtilis* exhibit high affinities toward the same DnaA box sequence and were found to interact in heterologous systems *in vitro*, creating similar oligomeric structures as was visualized by EM (Krause et al., 1997). However, neither was found to trigger open-complex formation on the heterologous origin. This provides further evidence that even when there are apparent similarities, the DnaA-*oriC* systems of individual species are not easily interchangeable.

Such observations may reflect that the mode through which DnaA interacts with particular boxes can differ among bacterial organisms. For example, the *E. coli* DnaA protein interacts efficiently with single, double or multiple DnaA boxes (Weigel et al., 1997; Speck and Messer, 2001). In contrast, the DnaA proteins of some other organisms have been found to strongly prefer two or more boxes over a single box. For example, the *M. tuberculosis* DnaA does not interact with a single box, while the closely related *S. coelicolor* DnaA interacts only weakly with a single box (Zawilak-Pawlik et al., 2005). Similarly, the *H. pylori* DnaA protein has a higher affinity for two boxes vs. a single box (Zawilak et al., 2003). Such observations suggest that the joint action of multiple DnaA monomers may be required for efficient binding in many cases. This is especially true for longer origins, in which a greater number of boxes appears to correlate with an increased importance of cooperative interactions among multiple DnaA monomers, such as suggested for the origins of the Actinomycetes – *M. tuberculosis* and *S. coelicolor* (Zawilak-Pawlik et al., 2005). The affinity of individual DnaA boxes can also vary within and across bacterial origins, with low-, medium-, and high-affinity DnaA boxes present in the origins. Interestingly, the number of low-affinity boxes often exceeds the number of high-affinity sites, such as seen in the *oriC* regions of *E. coli* and *C. crescentus* (Figures 1, 2; Rozgaja et al., 2011). Studies in *E. coli* showed that the number and distribution of low- and high-affinity sites is crucial for the activity of the *oriC* region, including its ability to control the frequency of initiation (Grimwade et al., 2007; Leonard and Grimwade, 2011). Low-affinity DnaA boxes provide a scaffold for DnaA oligomerization, whereas the high-affinity boxes (R1, R2, and R4; Figure 2) are believed to provide nucleation sites for the DnaA molecules. Low affinity-sites in *E. coli oriC* are organized into two oppositely oriented arrays separated by box R2 and flanked by boxes R1 and R4, which act as nucleation sites for DnaA oligomers (Rozgaja et al., 2011). The two DnaA oligomers were proposed to be extended by sequential interactions of DnaA monomers with arrayed low-affinity sites to finally form a contiguous DnaA filament (Rozgaja et al., 2011). It was suggested that such mode of a DnaA oligomer formation is directly implicated in origin unwinding since the two arrays of low-affinity sites are not helically phased and connection of the two halves of the oligomer would require specific twisting of the DNA strand, which would create a torsional stress (Rozgaja et al., 2011). It is worth noting that at least some of the origins of other bacteria also exhibit particular orientations of clusters of boxes (e.g., all *H. pylori* boxes share the same orientation) (Figure 2), which might indicate sequential binding of DnaA molecules and organized formation of a DnaA oligomer, as in *E. coli*.

The *E. coli* high-affinity boxes (R1, R2, and R4; Figure 1) appear to be occupied for the majority of the cell cycle, regardless of the nucleotide state of DnaA. The low-affinity boxes, on the other hand, are preferentially bound by ATP-DnaA (Miller et al., 2009; Rozgaja et al., 2011). The nucleotide state of DnaA is subjected to complex regulation system by RIDA inactivation, DARS-reactivation and rejuvenation as well as *de novo* protein synthesis (for details see Katayama et al., 2010; Leonard and Grimwade, 2011; Kasho et al., 2014) The binding of DnaA to low-affinity sites is additionally facilitated by the DiaA protein, which has been shown to stimulate the assembly of specific ATP-DnaA-*oriC* complexes (Keyamura et al., 2007), as well as specific *oriC*-binding proteins like SeqA, IHF and Fis (see “*oriC* activity is regulated by specific origin-binding proteins”).

The DUE region is a typically AT-rich stretch of nucleotides (comprehensively reviewed by Rajewska et al., 2012) that often includes characteristic repeated AT-rich sequences (e.g., that of *E. coli* comprises three 13-mer repeats) separated by short, non-AT-rich insertions. DUE regions are thermodynamically unstable compared to their neighboring sequences, rendering them susceptible to superhelical stress arising from the formation of the DnaA oligomer (Erzberger et al., 2006). The initially unwound region ranges from 20 to 60 bps in size, depending on the organism, which seems to provide sufficient space to accommodate a replicative helicase, DnaB (Figure 1) (Sutton et al., 1998; Abe et al., 2007; Mott et al., 2008; Keyamura et al., 2009). After the initial unwinding in *E. coli*, DnaA binds to single-stranded six-mer ATP-DnaA boxes (6-mer ssATP-DnaA boxes; Figure 1) located in the DUE (showing a strong preference for one of the strands), thereby stabilizing the initiation bubble prior to helicase loading (Speck and Messer, 2001). The bacterial DUE regions are always located upstream or downstream one or more DnaA box cluster(s), never in the midst of a cluster. It is important to note that the distance between the DUE and its proximal DnaA-box cluster is critical, as even slight changes were found to inhibit *oriC* unwinding (Hsu et al., 1994).

In sum, the existing evidence clearly shows that the cognate DnaA protein and DnaA boxes coevolved to achieve an optimal level of interaction. The orientation and spacing of DnaA boxes are both important for proper activity of the origin. For example, a change in the length of one helical turn between selected boxes does not affect initiation, but changes corresponding to part of a helical turn are highly detrimental (Woelker and Messer, 1993). At the level of an entire *oriC* region, the arrangement of individual boxes that differ in their affinities generates a specific order and assembly rate for the DnaA oligomer, which unwinds DNA in a precisely selected region called the DUE. From there, initiation events are further controlled by regulatory proteins that bind *oriC* at specific sites, as discussed below.

## *oriC* activity is regulated by specific origin-binding proteins

Transmission of genetic material to nascent cells requires precise regulation of chromosome replication and its coordination with the cell cycle. Since chromosomal replication is mainly regulated at the initiation stage, the principal activity of the *oriC* region (i.e., unwinding DNA) is tightly controlled. The relevant protein regulators are primarily involved in controlling the initial assembly of the DnaA oligomer along the origin of replication. The formation of an active orisome depends on the presence of proteins that: (i) regulate DnaA protein activity (e.g., Hda, which regulates the nucleotide-bound state of DnaA); (ii) facilitate the interactions between DnaA monomers (e.g., DiaA, which facilitates the assembly of the DnaA oligomer); or (iii) bind *oriC* and modulate the interaction of the DnaA protein with the origin of replication (Katayama et al., 1998; Kato and Katayama, 2001; Keyamura et al., 2007). In this section, we focus on various sequences that are targeted by the origin binding proteins (oriBPs) (other than DnaA) (Table 2) regulating the cell-cycle timing of replication from the *oriC* region (called “oriBP regulators”).

**Table 2:** **OriBP (origin binding protein) regulators**.

**OriBP**	**Binding sequence and features**	**Reference**
***ESCHERICHIA COLI***		
**SeqA**	5′-GATC-3′	Slater et al., 1995; Brendler et al., 1995; Taghbalout et al., 2000; Nievera et al., 2006
*(sequestration factor A)*	Binds to *oriC* and inhibits DnaA binding at low affinity sites.	
**Fis**	5′-GAACAACAGTTGTTC-3′	Gille et al., 1991; Filutowicz et al., 1992; Ryan et al., 2004
*(factor for inversion stimulation)*	Binds to single site in *oriC* and inhibits DnaA binding.	
**IHF**	5′-GATCAACAACCTG-3′ Binds to single site in *oriC*, stimulates DnaA binding at low affinity sites.	Filutowicz and Roll, 1990; Polaczek, 1990; Grimwade et al., 2000
*(integration host factor)*		
**HU**	Binds non-specifically to *oriC* and influences: DnaA oligomer stability at *oriC*, IHF binding, and stability of ds DNA helix.	Bonnefoy and Rouvière-Yaniv, 1992; Hwang and Kornberg, 1992; Ryan et al., 2002
*(histone like U-factor)*	Interacts with N-terminus of DnaA.	
**Dps**	Binds non-specifically to *oriC* and interacts with N-terminus of DnaA.	Chodavarapu et al., 2008b
*(DNA-binding protein from starved cells)*		
**ArcA**	Binds to 13-mer AT-rich repeats, and to DnaA, IHF, IciA binding sites in *oriC*.	Lee et al., 2001
*(aerobic respiration control)*	Influences DnaA interaction with AT-rich region.	
**IciA**	Binds to 13-mer AT-rich repeats in *oriC* and inhibits unwinding.	Hwang and Kornberg, 1990; Thöny et al., 1991; Hwang et al., 1992
*(inhibitor of chromosomal initiation)*		
**CspD**	No apparent target sequence, binds exclusively to ssDNA. Inhibits replication initiation.	Yamanaka et al., 2001
*(stationary phase-induced, stress response protein in the CspA family)*		
**Rob**	5′-ATCGCACGATCTGTATACTT-3′	Skarstad et al., 1993; Martin et al., 1999
*(right oriC-binding)*	Binds to single site in *oriC*. No clear function in initiation regulation.	
**H-NS**	5′-ATGATCGGTGATCCTG-3′	Kim et al., 2005; Yun et al., 2012a,b
*(heat-stable nucleoid structural protein)*	Binds to single site in *oriC*. Probably requires both Cnu and/or Hha proteins to bind DNA.	
**DpiA**	Binds to 13-mer AT-rich repeats in *oriC*. No function in initiation regulation.	Miller et al., 2003
*(destabilizer of plasmid inheritance)*		
***BACILLUS SUBTILIS***		
**Spo0A**	5′-TG[TA]CGAA-3′	Strauch et al., 1990; Castilla-Llorente et al., 2006
*(sporulation specific sigma factor)*	Binds to *oriC* and inhibits DnaA binding.	
***CAULOBACTER CRESCENTUS***		
**CtrA**	5′-TTAA[Nx7]TTAA-3′	Siam and Marczynski, 2000; Taylor et al., 2011
*(cell transcriptional regulator)*	Binds to *Cori* and inhibits DnaA binding.	
**IHF**	5′-TAACGCTCTGTT-3′	Siam et al., 2003
*(integration host factor)*	Binds to single site in *Cori*, displaces CtrA, which facilitates bending of DNA and promotes chromosome replication.	
***HELICOBACTER PYLORI***		
**HP1021**	5′-TGTT[TA]C[TA]-3	Donczew et al., 2014a
atypical response regulator	Binds to *oriC1* and *oriC2* and blocks DNA unwinding at the DUE (within the *oriC2*).	
***STREPTOMYCES COELICOLOR***		
**AdpA**	5′-TGGCSNGWWY-3′	Wolański et al., 2012
*(A-factor dependent protein)*	Binds to *oriC* and inhibits DnaA binding.	
***MYCOBACTERIUM TUBERCULOSIS***		
**IciA** **(Rv1985c)**	Binds to AT-rich region within *oriC* and blocks DNA unwinding.	Kumar et al., 2009
*(inhibitor of chromosomal initiation)*		
**MtrA**	5′-GTCACAGCG-3′ Mechanism not known.	Rajagopalan et al., 2010
*(Mycobacterium tuberculosis response regulator A)*		

The oriBP regulators can be divided into three classes depending on their target sequences: (i) those that interact with DnaA boxes or in their close vicinity; (ii) those that interact with AT-rich sequences within the DUE; and (iii) those that interact with other sequences within *oriC* (Figure 1). The oriBPs can also be classified by their sequence specificity and/or function: they may specifically or non-specifically interact with *oriC* to positively or negatively influence the unwinding of the origin. They confer their direct effects by binding to DnaA (or other oriBPs) binding sites, and exert their indirect effects by changing the DNA structure of the origin to modulate the binding of additional oriBPs.

The proteins that regulate replication initiation have been best described for *E. coli*, in which ~11 *oriC* binding proteins have been identified (Table 2). However, we do not yet fully understand the roles played by all of these oriBPs in regulating replication. Here, we use the *E. coli* model to discuss the roles of particular oriBP regulators in the sequential events that are believed to occur following the initiation of replication. When possible, we also describe the roles of counterpart proteins in other bacteria and discuss alternative initiation regulators that are not found in *E. coli*.

In *E. coli*, shortly after chromosomal replication the SeqA protein binds to several sites within the *oriC* region to strictly prevent the initiation of new rounds of replication via a mechanism called “sequestration.” SeqA specifically binds the short palindromic sequence, GATC, which is overrepresented within *oriC* compared to the rest of the bacterial chromosome. Newly replicated origins are hemimethylated for about 1/3 of the *E. coli* cell cycle, and SeqA preferentially binds hemimethylated GATC sequences over the fully methylated sequences. Thus, SeqA sequesters the *oriC* region until the GATC sites are fully methylated by the Dam methylase (Campbell and Kleckner, 1990; Lu et al., 1994; Brendler et al., 1995; Slater et al., 1995). SeqA predominantly inhibits replication initiation by blocking DnaA from binding to the R5, I2, I3, τ1, and τ2 sites, which overlap with the GATC sequences (Taghbalout et al., 2000; Nievera et al., 2006). This prevents the DnaA filament from being elongated from the high-affinity DnaA boxes, R1, R2, and R4, although it does not alter their occupation by DnaA (Samitt et al., 1989; Cassler et al., 1995; Nievera et al., 2006). This sequestration mechanism appears to be exclusive to a few DamMT-specifying proteobacteria, as homologs of the *seqA* gene have been identified only in this subset of Gram-negative bacteria (Brézellec et al., 2006).

Another negative regulator of initiation in *E. coli*, the Fis protein, associates with *oriC* throughout most of the cell cycle; similar to SeqA, Fis negatively influences replication initiation by regulating the occupation of DnaA on low-affinity sites (Cassler et al., 1995; Ryan et al., 2004). Fis specifically binds to a single site that is located between R2 and R3, and overlaps with the C3 DnaA binding site (Figure 1) (Gille et al., 1991; Filutowicz et al., 1992). Fis binding is thought to competitively inhibit the interaction of DnaA with this region (Ryan et al., 2004), and Fis exhibits a DNA-bending activity that plays a yet-unknown role (Finkel and Johnson, 1992; Ryan et al., 2004).

In addition to competing with DnaA for binding to *oriC*, both Fis and SeqA also negatively regulate the interaction of another oriBP, IHF, with the origin. In contrast to the former two proteins, IHF positively regulates replication initiation (Hwang and Kornberg, 1992; Grimwade et al., 2000; Ryan et al., 2002). As the time of initiation draws near, increasing levels of DnaA trigger the displacement of Fis and the full methylation of DNA weakens SeqA binding, ending the repressive activities of these proteins (Slater et al., 1995; Ryan et al., 2004). The release of SeqA reveals the IHF binding site; displacement of Fis promotes IHF binding; and IHF binding leads to bending of the DNA (Polaczek, 1990; Cassler et al., 1995; Rice et al., 1996; Weisberg et al., 1996; Swinger and Rice, 2004). IHF then stimulates the binding of DnaA-ATP to low-affinity sites (thus redistributing the DnaA protein) and induces the unwinding of *oriC* (Grimwade et al., 2000). Notably, the transcription of the *dnaA* gene is also subject to regulation by the SeqA protein (Campbell and Kleckner, 1990; Theisen et al., 1993; Bogan and Helmstetter, 1997). Thus, the increased DnaA concentrations that trigger the displacement of Fis displacement presumably reflect the earlier release of the *dnaA* promoter from inhibition by SeqA. In *C. crescentus*, the protein that corresponds to IHF also binds to a single site within the *oriC* of this species (*Cori*). Here, the recognition sequence for IHF overlaps the C-binding site for CtrA, which negatively regulates chromosomal replication in *C. crescentus* (for more on CtrA, see below). In this system, IHF binding leads to the displacement of CtrA from *Cori*, allowing the DNA to bend and promoting replication (Siam et al., 2003).

In *E. coli*, HU is a second positive regulator of initiation. Although this histone-like protein was believed to non-specifically bind DNA, some evidence has suggested that it may interact with *oriC* in a specific manner (Bonnefoy and Rouvière-Yaniv, 1992; Ryan et al., 2002). HU enhances the DnaA-dependent unwinding of *oriC*. This presumably occurs through its ability to bend and destabilize DNA (Hwang and Kornberg, 1992; Ryan et al., 2002). However, HU was further shown to interact with the N-terminal part of DnaA to stabilize the DnaA oligomer assembled at *oriC* (Chodavarapu et al., 2008a), suggesting that *oriC* unwinding may also be stimulated through this additional mechanism. Interestingly, HU was also shown to reduce the binding of DnaA at the DnaA-I3 site (Ryan et al., 2002), and modulate the binding of IHF to *oriC* in a manner dependent on the relative concentrations of IHF and HU (Bonnefoy and Rouvière-Yaniv, 1992).

The oriBPs, Dps, and ArcA, negatively regulate replication initiation in response to oxidative stress and oxygen depletion, respectively (Almirón et al., 1992; Lee et al., 2001; Chodavarapu et al., 2008b). Dps non-specifically binds DNA and interacts with the N-terminus of the DnaA protein to inhibit DNA unwinding (Almirón et al., 1992; Chodavarapu et al., 2008b). It has been suggested that Dps may act as a checkpoint during oxidative stress, delaying initiation until the oxidative DNA damage has been repaired (Chodavarapu et al., 2008b). Under anaerobic conditions, in contrast, ArcA is phosphorylated by a cognate kinase of the two-component system. ArcA-P transcriptionally regulates the genes required to maintain anaerobic growth (Lee et al., 2001), and it is also thought to regulate the activity of *oriC*. *In vitr*o, ArcA-P binds a region that contains AT-rich 13-mers and the binding sites for IHF and DnaA (R1 box). It prevents the formation of the open complex without displacing IHF or DnaA from the DNA (Lee et al., 2001), suggesting that ArcA-P may disrupt the interaction between the DnaA protein and the AT-rich region.

Interestingly, ArcA-P is capable of displacing another oriBP, IciA, which specifically binds to the 13-mer AT-rich region and inhibits the unwinding of *oriC* (Hwang and Kornberg, 1990, 1992; Thöny et al., 1991). Interestingly, IciA is also capable of transcriptionally regulating genes known to be involved in DNA replication (e.g., *dnaA*) and amino acid metabolism (Lee et al., 1996; Nandineni and Gowrishankar, 2004; Bouvier et al., 2008). A study of the IciA counterpart in *Mycobacterium tuberculosis* showed that this protein also binds to the AT-rich region of the *oriC* and *in vitro* blocks DnaA-dependent helix opening, and may play a role in maintaining mycobacterial latency (during which DNA replication is arrested) (Kumar et al., 2009).

Regarding additional oriBPs in *E. coli*, the CspD protein reportedly inhibits both the initiation and elongation of chromosomal replication *in vitro* (Yamanaka et al., 2001). Finally, additional proteins capable of specifically binding *oriC* have been identified and described (e.g., Rob, H-NS, and DpiA), but their specific roles and contributions to the replication initiation process are not yet known (Skarstad et al., 1993; Martin et al., 1999; Miller et al., 2003; Kim et al., 2005; Yun et al., 2012a,b).

In bacteria that undergo a complex life cycle (e.g., *Bacillus, Caulobacter*, and *Streptomyces*), the regulation of replication initiation must also be adjusted to the developmental stage to ensure that each nascent cell receives a single copy of the chromosome (Wolański et al., 2014). Recently, master transcription factors known to regulate the expression levels of hundreds of genes involved in cell cycle progression and cell differentiation were demonstrated to be also involved in controlling frequency of chromosomal replication initiation events. Examples of these are Spo0A, CtrA, and AdpA proteins, which temporally and spatially coordinate chromosome replication with developmental program in *B. subtilis, C. crescentus*, and *S. coelicolor*, respectively (Laub et al., 2000, 2002; Molle et al., 2003; Fujita and Losick, 2005; Fujita et al., 2005; Ohnishi et al., 2005; Wolański et al., 2011). They bind specifically to relevant recognition sequences within the origin of replication and inhibit the binding of DnaA, thereby disrupting assembly of the DnaA oligomer and inhibiting replication initiation (Siam and Marczynski, 2000; Castilla-Llorente et al., 2006; Taylor et al., 2011; Wolański et al., 2012; Boonstra et al., 2013; reviewed in Wolański et al., 2014). In all three cases, the binding sites for these regulators overlap with one or more DnaA binding sites, setting up a competition between the regulator and initiator for binding to *oriC* (Figure 2). Interestingly, the activities of Spo0A and CtrA are regulated by phosphorylation, which enhances their binding to DNA. Increasing the levels of these active proteins inhibits chromosomal replication and stimulates the expression levels of various genes responsible for differentiation.

In the pathogenic *Mycobacterium, M. tuberculosis*, in addition to IciA, the MtrA protein has been shown to bind the *oriC* region and regulate chromosomal replication (Rajagopalan et al., 2010). MtrA binds specifically to four MtrA boxes that are dispersed throughout the *oriC*, between the DnaA boxes (Figure 2). Each MtrA box consists of two direct repeats of GTCACAgcg-like sequences. Mutations in the MtrA binding sequences were found to compromise the replication of the minichromosome (an *oriC* containing plasmid), whereas increased levels of MtrA appear to be associated with deficient autonomous replication of the minichromosome (Rajagopalan et al., 2010). Thus, MtrA may play both positive and negative roles in the initiation of replication. The exact action mechanism of MtrA at *oriC* is not yet known, but it has been suggested that this protein may facilitate or hinder the ability of DnaA to oligomerize at *oriC*, rather than interfering with the direct binding of the initiator protein. MtrA has been identified as a response regulator component of the signal transduction system, MtrAB, which suggests that its role in replication initiation might depend on its phosphorylation status (Via et al., 1996; Fol et al., 2006; Rajagopalan et al., 2010). Interestingly, it has been recently shown that in other pathogenic bacterium, *H. pylori*, the orisome assembly is controlled by HP1021 protein – the orphan response regulator, which was previously shown to affect expression of nearly 80 genes (Pflock et al., 2007). HP1021 competes with DnaA for the binding sites at *oriC* and inhibits DNA unwinding at the DUE site (Donczew et al., 2014a). It suggests that HP1021 controls initiation of *H. pylori* chromosome replication in response to yet unknown stimuli. It is very likely that in numerous bacteria chromosome replication is regulated by signal transduction systems in response to cellular or external stimuli affecting bacterial growth.

## Conclusion and outlook

In sum, the bacterial origins differ across organisms in the organization of their DNA modules, but all origins encode comprehensive instructions for the assembly and disassembly of the orisome-forming proteins, enabling the timely regulation of this first and crucial step in chromosomal replication. The instructions direct the sequential binding of DnaA molecules to the available array of high- and low-affinity DnaA boxes to form a nucleoprotein complex that triggers the unwinding of DNA within the AT-rich region of the *oriC*. The *oriC*-encoded instructions also guide a number of other *oriC*-binding proteins that directly or indirectly respond to environmental signals and induce or repress formation of the DnaA-*oriC* complex, thereby modulating replication initiation. Tight regulation of the initiation process is achieved in all bacteria, albeit via different strategies involving various *oriC* binding proteins, many of which play additional roles in cell-cycle regulation. In pathogens, the functions of some initiation regulators may also depend on interactions with the host cell cycle; however, such interactions have not yet been thoroughly elucidated. It is important to remember that origins do not contain universal instructions. Only origins from very closely related organisms exhibit similar organizations, and the repertoire of regulatory proteins is unique for each species or group of related organisms. That enables a bacterium to perfectly adjust its replication to the cell cycle and coordinate its growth with external stimuli. As reviewed herein, we know a great deal about origins and their structures. To continue progressing in this field, we need detailed analyses of orisome formation, as has already been done for *E. coli* and (to a lesser extent) a limited number of other organisms (e.g., *B. subtilis* or *M. tuberculosis*). Future studies should examine how differences in origin structure are translated to the species-specific characteristics of DnaA oligomerization and its control by regulatory proteins. In addition, many important aspects of the replication initiation process remain to be discovered, particularly in pathogens, including the answers to questions, such as:

How is replication initiation coordinated with the cell cycles of different pathogens upon host infection?
How do host signals modulate or influence replication initiation in pathogenic bacteria?

### Conflict of interest statement

The authors declare that the research was conducted in the absence of any commercial or financial relationships that could be construed as a potential conflict of interest.
